# (Poly)phenols: The Missing Piece in the Puzzle of Inflammation

**DOI:** 10.3390/ijms242316971

**Published:** 2023-11-30

**Authors:** Inês Figueira, María Ángeles Ávila-Gálvez, Natasa Loncarevic-Vasiljkovic, Cláudia Nunes dos Santos

**Affiliations:** NOVA Medical School, Faculty of Medicine, NOVA University of Lisbon (NMS|FCM), 1150-082 Lisbon, Portugal; ines.figueira@nms.unl.pt (I.F.); angela.avila@nms.unl.pt (M.Á.Á.-G.); natasa.loncarevic@nms.unl.pt (N.L.-V.)

Despite researchers’ and clinicians’ exponential understanding of chronic diseases’ complexity, ranging from cancer, diabetes, and neurodegenerative disorders, we still have a lot of unanswered questions on pathobiology mechanisms, wherein inflammation is central.

Inflammation constitutes a physiological machinery wherein the immune system identifies and eliminates detrimental and foreign stimuli [1]. This process, occurring in either an acute (i.e., within physiological conditions) or chronic (i.e., in a disease scenario) manner, involves the activation of cellular and molecular events aiming to minimize injury or combat infection during acute inflammatory responses. Successful execution of these mechanisms contributes to the restoration of homeostasis and the resolution of acute inflammation [2]. Nevertheless, in instances wherein such protective mechanisms falter, or acute inflammation persists and transitions to a chronic state, the groundwork for the onset of chronic inflammatory diseases is laid [2]. It is therefore logical to consider the management of inflammation as a reliable and solid approach to tackling chronic incurable disorders, both via preventive and/or therapeutic approaches.

(Poly)phenols, which are phytochemicals abundant in several fruits, vegetables, and beverages, are widely acknowledged as powerful anti-inflammatory molecules [3]. The scientific community has successfully explored and dissected the multifaceted roles played by these compounds in modulating inflammatory responses. In the nutritional context, intake of (poly)phenols through the diet and adherence to particular dietary patterns (such as the Mediterranean diet) have been linked to the prevention of chronic inflammatory diseases [4,5]. Mechanistic studies have been also fundamental to growing our understanding of the precise mechanisms of action of (poly)phenols and their physiologically relevant metabolites (microbial phenolic metabolites, as well as their phase-II-derived metabolites) in inflammatory processes. From elucidating cellular signaling pathways to unraveling the molecular mechanisms underlying the anti-inflammatory properties of (poly)phenols and their bioavailable metabolites [6,7], contributions in recent years have been both enlightening and transformative.

One of the most compelling aspects of such research has been the convergence of evidence pointing toward (poly)phenols as pivotal components in the “inflammatory puzzle”. Whether sourced from fruits, vegetables, or other natural reservoirs, ranging from pharmacological approaches to nutritional ones, these bioactive compounds have demonstrated remarkable potential in mitigating inflammatory processes at various levels [8,9].

The translational implications of (poly)phenol research have become increasingly evident. As we navigate the complex landscape of chronic inflammatory diseases, the incorporation of (poly)phenol-rich interventions into preventive and therapeutic strategies holds promise. The prospect of dietary modifications, nutraceutical supplementation, or the development of targeted pharmaceutical agents based on (poly)phenol frameworks represent an exciting frontier for future investigations.

Nevertheless, with every answer comes a cascade of new questions. In this Special Issue, we have made significant strides in unraveling the intricacies of inflammation within (poly)phenolic compounds, with solid revision works on their potential against traumatic brain injury [10] and in wound healing [11], as well as against inflammation cascades observed in triple-negative breast cancer [12] or upon lipopolysaccharide exposure [13]. Nevertheless, much remains to be explored. The nuances of (poly)phenol interactions with specific cell types, the impact of different chemical structures on bioactivity, and the individual responses to (poly)phenol interventions are among the intriguing avenues that beckon further inquiry.

Altogether, this Special Issue serves not only as proof of the current state of (poly)phenol research in the context of inflammation, but also as a catalyst for future explorations. Multidisciplinary approaches, the integration of cutting-edge technologies, and the sustained curiosity that drives scientific inquiry will undoubtedly propel us closer to unlocking the full potential of (poly)phenols within the intricate puzzle of inflammation.
